# Notch2 Signaling Drives Cardiac Hypertrophy by Suppressing Purine Nucleotide Metabolism

**DOI:** 10.34133/research.0635

**Published:** 2025-03-18

**Authors:** Yuhong Wang, Yizhe Li, Shihong Chen, Tingting Yu, Weiyan Sun, Jiao Liu, Huiwen Ren, Yao Zhou, Lu Wang, Xixi Tao, Ronglu Du, Wenlong Shang, Yinxiu Li, Danyang Tian, Bei Wang, Yujun Shen, Qian Liu, Ying Yu

**Affiliations:** ^1^ Department of Pharmacology, Tianjin Key Laboratory of Inflammatory Biology, Center for Cardiovascular Diseases, Key Laboratory of Immune Microenvironment and Disease (Ministry of Education), The Province and Ministry Co-sponsored Collaborative Innovation Center for Medical Epigenetics, State Key Laboratory of Experimental Hematology, School of Basic Medical Sciences, Tianjin Medical University, Tianjin, China.; ^2^ Department of Cardiology, Tianjin Medical University General Hospital, Tianjin Medical University, Tianjin, China.

## Abstract

Gain-of-function mutations of Notch2 cause the rare autosomal dominant disorder known as Hajdu–Cheney syndrome (HCS). Most patients with HCS develop congenital heart disease; however, the precise mechanisms remain elusive. Here, a murine model expressing the human Notch2 intracellular domain (hN2ICD) in cardiomyocytes (hN2ICD-Tg^CM^) was generated and the mice spontaneously developed ventricular diastolic dysfunction with preserved ejection fraction and cardiac hypertrophy. Ectopic hN2ICD expression promoted cardiomyocyte hypertrophy by suppressing adenylosuccinate lyase (ADSL)-mediated adenosine 5′-monophosphate (AMP) generation, which further enhanced the activation of the mammalian target of rapamycin complex 1 pathway by reducing AMP-activated kinase activity. Hairy and enhancer of split 1 silencing abrogated hN2ICD-induced cardiomyocyte hypertrophy by increasing Adsl transcription. Importantly, pharmacological activation of AMP-activated kinase ameliorated cardiac hypertrophy and dysfunction in hN2ICD-Tg^CM^ mice. The frameshift mutation in Notch2 exon 34 (c.6426dupT), which causes early-onset HCS, induces AC16 human cardiomyocyte hypertrophy through suppressing ADSL-mediated AMP generation. Thus, targeting Notch2-mediated purine nucleotide metabolism may be an attractive therapeutic approach to heart failure treatment.

## Introduction

Hajdu–Cheney syndrome (HCS) is an inherited disease associated with gain-of-function mutations in the Notch2 gene [1]. Many patients with HCS also have congenital heart diseases, such as atrial and ventricular septal defects and patent ductus arteriosus, and require cardiac surgery [2]. The frameshift mutations in exon 34 of Notch2 create a short protein product lacking the proline, glutamic acid, serine, and threonine (PEST) domain, which is resistant to ubiquitin-mediated degradation and causes gain-of-Notch2 function [3,4]. Although Notch2 has a structure similar to that of other Notch family members (Notch1, Notch3, and Notch4), it exhibits distinct functions during embryonic development of the heart. Animal experiments have revealed that Notch2 is required for atrioventricular canal development [5] and proper formation of the heart outflow tract in mice [6]. Unlike Notch1, whose activity is primarily limited to the endocardium [7], Notch2 is expressed in the myocardium during murine heart development [8]. Hypomorphic mutations in Notch2 result in myocardial hypoplasia and reduced trabeculation [9], whereas constitutively active Notch2 in the myocardium leads to hypertrabeculation and ventricular septum defects in mice [10]. However, the precise molecular mechanisms underlying Notch2-related cardiac dysfunction remain unclear.

The Notch signaling pathway regulates cell fate specification and modulates organ development and homeostasis. The Notch intracellular domain is released when Notch receptors bind to the specific ligands, then it translocates to the nucleus, forms a transcriptional activator complex with recombining binding protein suppressor of hairless, and triggers the production of target genes, including *Hey* and *Hes* [11]. Accumulated evidence has shown that Notch signaling is also a crucial regulator of cellular energy homeostasis and metabolism in various cell and tissue types [12]. For instance, activation of Notch induces glycogenolysis in hepatocytes and increases the rate of glucose uptake in muscles [13]. Adipose-specific inactivation of Notch1 leads to browning of white adipose tissue by elevating uncoupling protein 1 expression [14]. Notch-driven T-cell acute lymphoblastic leukemia utilizes ubiquitin protein ligase E3 component n-recognin 7 to enhance nucleotide biosynthesis and maintain oncogenic potential [15]. Inhibition of Notch in the endothelium causes cardiac hypertrophy and failure through the suppression of fatty acid metabolism [16]. Notably, the development of heart hypertrophy and failure are linked to abnormalities of heart energy metabolism [17,18]. However, whether Notch signaling influences cardiac function by modulating cell metabolism has not yet been explored.

In this study, we introduced the human Notch2 intracellular domain (hN2ICD) into mouse cardiomyocytes (CMs) to model the cardiac defects in patients with HCS and found that overexpression of hN2ICD in cardiomyocytes led to cardiac hypertrophy and left ventricular diastolic dysfunction with preserved ejection fraction (EF) in mice, resembling heart failure with preserved ejection fraction (HFpEF) in humans. An overdose of hN2ICD led to cardiomyocyte hypertrophy by suppressing the expression of adenylosuccinate lyase (ADSL), a key enzyme in purine nucleotide biosynthesis, which resulted in low intracellular adenosine 5′-monophosphate (AMP) generation, decreased AMP-activated kinase (AMPK) activity, and subsequently enhanced mammalian target of rapamycin (mTOR) complex 1 signaling. We identified the Notch2 target gene hairy and enhancer of split 1 (Hes1) as a transcriptional repressor of Adsl in hypertrophic cardiomyocytes. Treatment with aminoimidazole carboxamide riboside (AICAR), a direct AMPK agonist, markedly ameliorates cardiac dysfunction in hN2ICD-Tg^CM^ mice. Notch2 gain-of-function mutation in exon 34 (c.6426dupT) recapitulates pathological hypertrophy in a human cardiomyocyte cell line by disrupting AMP production. Thus, excessive Notch2 signaling promotes cardiac hypertrophy and dysfunction by modulating purine nucleotide metabolism.

## Results

### CM-specific overexpression of hN2ICD leads to cardiac hypertrophy and diastolic dysfunction in mice

**Fig. 1.**
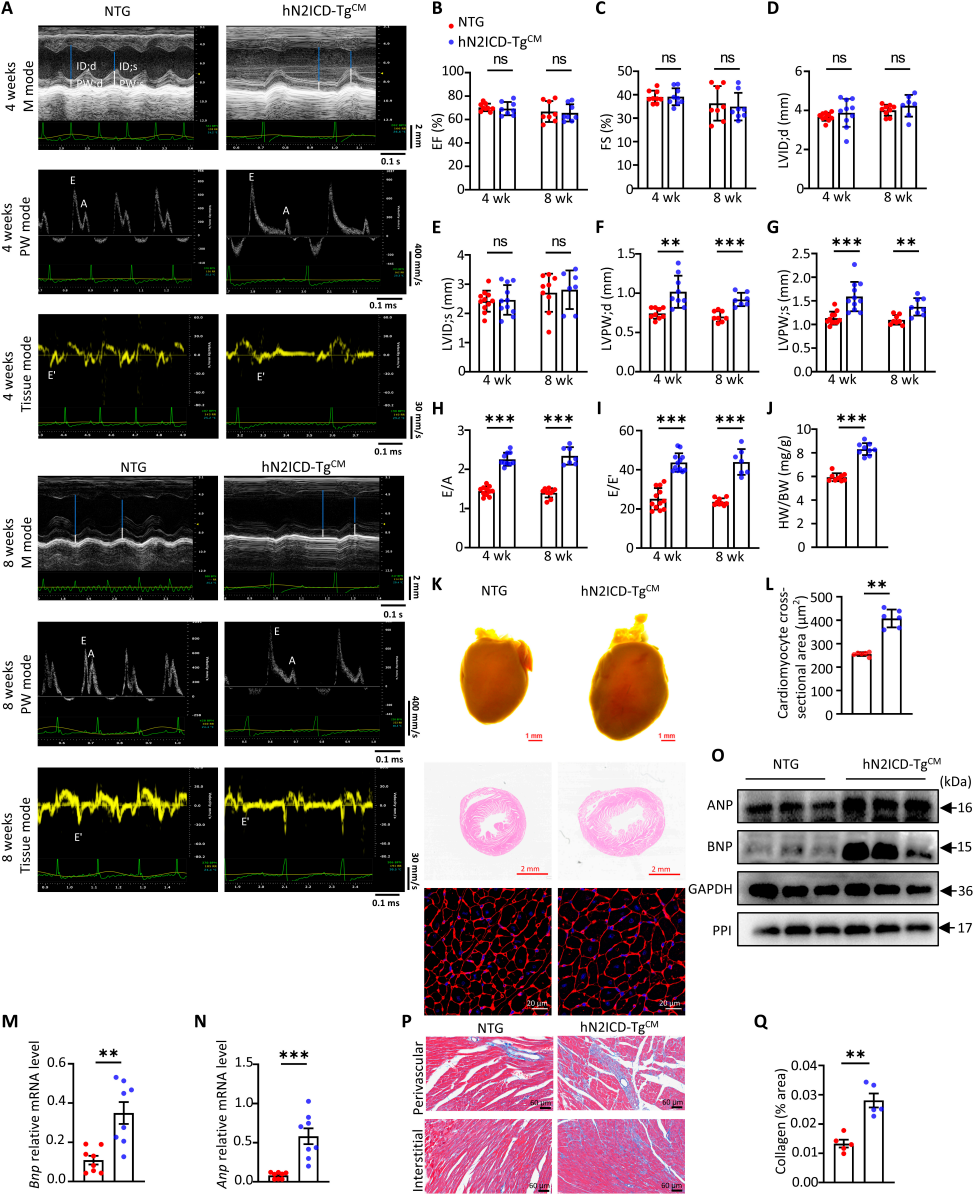
Mice with the human Notch2 intracellular domain (hN2ICD) overexpressed in cardiomyocytes spontaneously develop cardiac hypertrophy and diastolic dysfunction. (A) Representative echocardiographic tracings of non-transgenic (NTG) and hN2ICD-Tg^CM^ mice. (B and C) Percentage of left ventricular ejection fraction (LVEF) and left ventricular fractional shortening (LVFS). *n* = 8 and 9. (D and E) Left ventricular internal dimensions at end diastole (LVID;d) and end systole (LVID;s) were obtained from echocardiography. *n* = 7 to 11. (F and G) Analysis of left ventricular posterior wall thicknesses at end diastole (LVPW;d) and end systole (LVPW;s). *n* = 7 to 11. (H) Ratio between E (early filling) and A (atrial filling) waves. *n* = 7 to 11. (I) Ratio between E and E′ waves (E/E′). *n* = 7 to 12. (J) The heart weight to body weight (HW/BW) ratio of mice. *n* = 9. (K) Representative images of whole-mount hearts and hematoxylin and eosin (H&E) and wheat germ agglutinin (WGA) stained sections of the hearts. Scale bars, 1 mm (general view), 2 mm (H&E), and 20 μm (WGA). *n* = 6. (L) Quantitative analysis of averaged cardiomyocyte cross-sectional area based on WGA staining. *n* = 6. (M and N) Relative messenger RNA (mRNA) levels of *Bnp* and *Anp* in mouse hearts. *n* = 8. (O) Relative protein of atrial natriuretic peptide (ANP) and brain natriuretic peptide (BNP) in mouse hearts. (P) Representative images of Masson’s trichrome staining. Scale bar, 60 μm. *n* = 5. (Q) Quantification of collagen content in (P). Statistical significance was evaluated by using the Student *t* test for (B), (C), (E), (H), and (J); Welch’s *t* test for (D), (F), (G), and (I); and the Mann–Whitney *U* test for (L), (M), (N), and (Q). ID;d, internal dimension at end diastole; ID;s, internal dimension at end systole; PW;d, posterior wall thicknesses at end diastole; PW;s, posterior wall thicknesses at end systole; PW, pulsed-wave; ns, not significant; EF, ejection fraction; FS, fractional shortening; GAPDH, glyceraldehyde-3-phosphate dehydrogenase; PPI, peptidylprolyl isomerase A.

To ascertain the role of Notch2 signaling in the regulation of cardiac function, cardiomyocyte-specific hN2ICD transgenic mice (hN2ICD-Tg^CM^) were created by crossing αMHC^Cre^ mice with hN2ICD-Tg^stop^ mice (non-transgenic [NTG]), in which the hN2ICD coding region was targeted in Rosa locus in mice (Fig. S1A). Genotyping, reverse transcriptase polymerase chain reaction (RT-PCR), Western blotting, and immunofluorescence analysis confirmed that hN2ICD was highly expressed in the cardiac tissues and cardiomyocytes of hN2ICD-Tg^CM^ mice (Fig. S1B to E). Ultrasound cardiac imaging examinations (Fig. 1A) revealed that hN2ICD-Tg^CM^ mice showed comparable left ventricular EF and fractional shortening (FS), and left ventricular internal dimensions at end diastole (LVID;d) and end systole (LVID;s) compared to those of littermate control mice at 4 and 8 weeks after birth (Fig. 1B to E). Notably, we found a significant increase in left ventricular posterior wall thicknesses at end systole (LVPW;s) and end diastole (LVPW;d) in hN2ICD-Tg^CM^ mice (Fig. 1F and G), accompanied by elevated E wave to A wave (E/A) and E wave to E′ wave (E/E′) ratios (Fig. 1H and I), indicating a phenotype of cardiac hypertrophy and diastolic dysfunction in mice. This notion was further confirmed by the elevated ratio of heart weight to body weight (HW/BW) (Fig. 1J), enlarged CM surface area (Fig. 1K and L), and elevated expression of cardiac fetal genes, such as brain natriuretic peptide (BNP) and atrial natriuretic peptide (ANP) (Fig. 1M to O) in 8-week-old hN2ICD-Tg^CM^ mice. Meanwhile, excessive cardiac collagen deposition was observed in hearts from 8-week-old hN2ICD-Tg^CM^ mice, indicating more severe fibrosis (Fig. 1P and Q). Taken together, the cardiomyocyte-specific overexpression of Notch2 intracellular domain (N2ICD) led to cardiac hypertrophy and an HFpEF-like phenotype in mice.

### hN2ICD overexpression promotes CM hypertrophy by disrupting purine metabolism

**Fig. 2.**
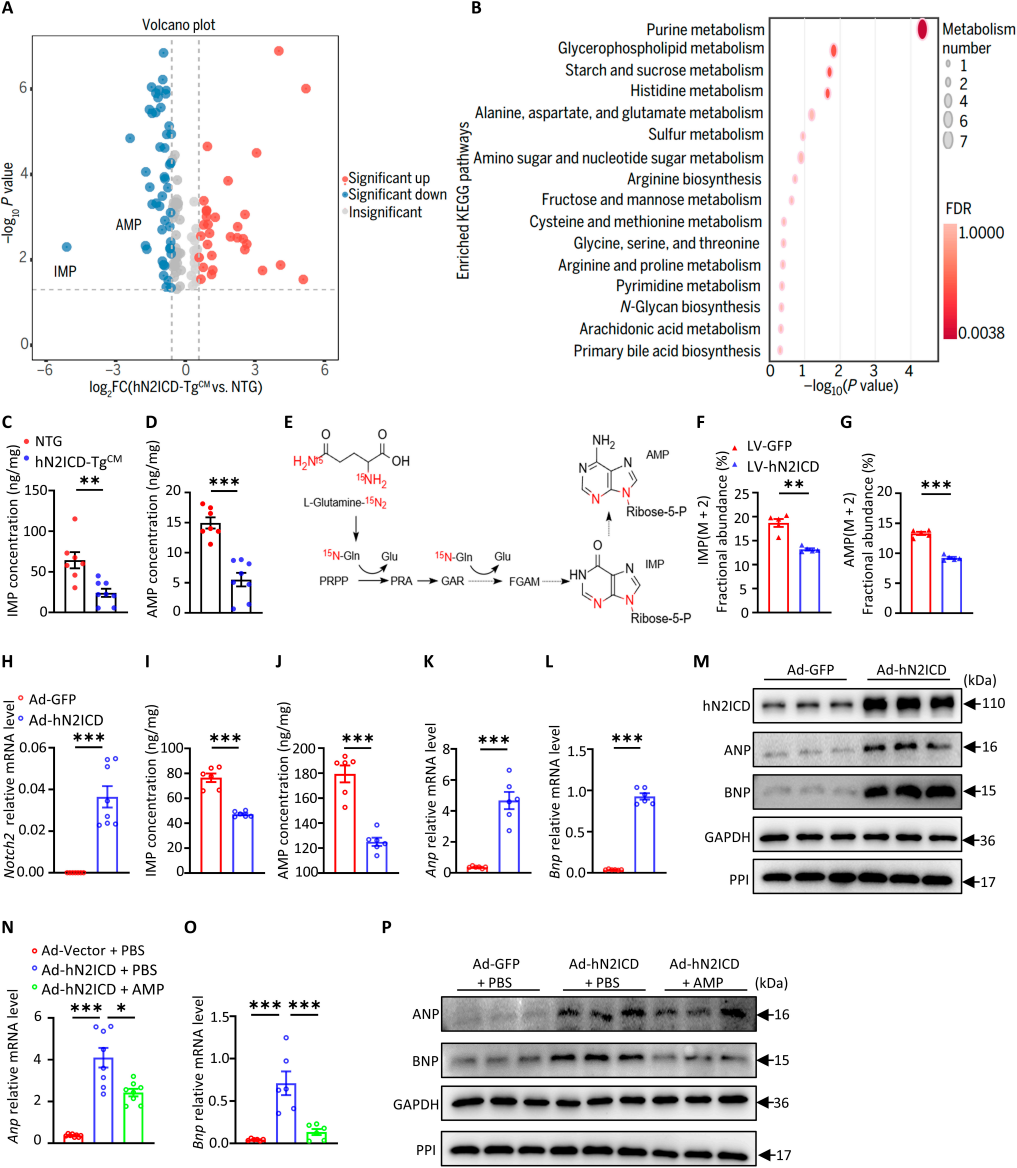
hN2ICD overexpression facilitates cardiomyocyte hypertrophy by compromising purine metabolism. (A) Volcano plot of the metabolic profile in mouse heart tissue (hN2ICD-Tg^CM^ vs. NTG). (B) Kyoto Encyclopedia of Genes and Genomes (KEGG) pathway enrichment analysis of differential metabolites. (C and D) Adenosine 5′-monophosphate (AMP) and inosine 5′-monophosphate (IMP) concentrations in mouse heart tissues were quantified using liquid chromatography–tandem mass spectrometry (LC–MS/MS). *n* = 7 and 8. (E) Schematic showing how nitrogen from glutamine is added to the purine ring. (F and G) Fractional abundance of ^15^N_2_-labeled IMP and AMP in AC16 cells labeled with ^15^N_2_-glutamine for 4 h. *n* = 5. (H) Relative mRNA levels of *Notch2* in neonatal rat ventricular myocytes (NRVMs) with or without hN2ICD overexpression. *n* = 7 and 8. (I and J) Intracellular IMP and AMP concentrations in NRVMs with or without hN2ICD overexpression. *n* = 6. (K and L) Relative mRNA levels of *Anp* and *Bnp* in NRVMs with or without hN2ICD overexpression. *n* = 6. (M) Western blot analysis of Notch2 intracellular domain (N2ICD), ANP, and BNP protein expression in NRVMs with or without hN2ICD overexpression. (N and O) Relative mRNA levels of *Bnp* and *Anp* in hN2ICD-overexpressed NRVMs with or without AMP treatment. *n* = 6 to 8. (P) Western blot analysis of the protein expression of ANP and BNP in hN2ICD-overexpressed NRVMs with or without AMP treatment. Statistical analyses were performed by using the Student *t* test for (C), (D), (G), and (J); Welch’s *t* test for (F), (I), (K), and (L); the Mann–Whitney *U* test for (H); Brown–Forsythe and Welch one-way analysis for (N); and one-way ANOVA followed by Tukey’s multiple-comparisons test for (O). FDR, false discovery rate; PRPP, phosphoribosyl pyrophosphate; PRA, phosphoribosylamine; GAR, glycineamide ribonucleotide; FGAM, formylglycinamidine ribonucleotide; ribose 5-P, ribose 5-phosphate; LV-GFP, lentivirus encoding green fluorescent protein; LV-hN2ICD, lentivirus encoding hN2ICD; Ad-GFP, adenovirus encoding green fluorescent protein; Ad-hN2ICD, adenovirus encoding hN2ICD; PBS, phosphate-buffered saline.

To investigate how hN2ICD modulates CM hypertrophy, we performed an untargeted metabolomic analysis of cardiac tissues from NTG and hN2ICD-Tg^CM^ mice using liquid chromatography–tandem mass spectrometry (LC–MS/MS) and identified 126 differentially expressed metabolites (least squares discriminant analysis (DA) variable importance projection scores > 1 and *P* < 0.05). Markedly, the purine metabolite inosine 5′-monophosphate (IMP) was significantly decreased in hN2ICD-Tg^CM^ hearts (Fig. 2A). Kyoto Encyclopedia of Genes and Genomes (KEGG) analysis recognized that differentially expressed metabolites were most enriched in purine metabolism (Fig. 2B), and targeted metabolomic analysis confirmed the decrease in IMP and AMP levels in hN2ICD-Tg^CM^ hearts (Fig. 2C and D). Next, we performed a stable isotope tracer experiment to further explore the role of hN2ICD on de novo purine biosynthesis in cardiomyocytes. hN2ICD-overexpressed human AC16 cardiomyocytes were treated with a 4-h pulse of stable-isotope-labeled ^15^N_2_-glutamine, which contributes to purine ring synthesis [19,20] (Fig. 2E). Similarly, the IMP and AMP products from ^15^N_2_-glutamine were also reduced in AC16 cells overexpressing hN2ICD (Fig. 2F and G). In addition, adenovirus-mediated hN2ICD overexpression (Fig. 2H and M) significantly reduced intracellular IMP and AMP production (Fig. 2I and J) and led to a hypertrophic phenotype in cultured neonatal rat ventricular myocytes (NRVMs), as evidenced by elevated ANP and BNP expression (Fig. 2K to M). AMP replenishment in cultured hN2ICD-overexpressed NRVMs abrogated the induction of ANP and BNP expression (Fig. 2N to P). Taken together, activation of Notch2 facilitates CM hypertrophy by interrupting AMP and IMP metabolism in CMs.

### hN2ICD drives CM hypertrophy by suppressing ADSL-mediated IMP and AMP synthesis

**Fig. 3.**
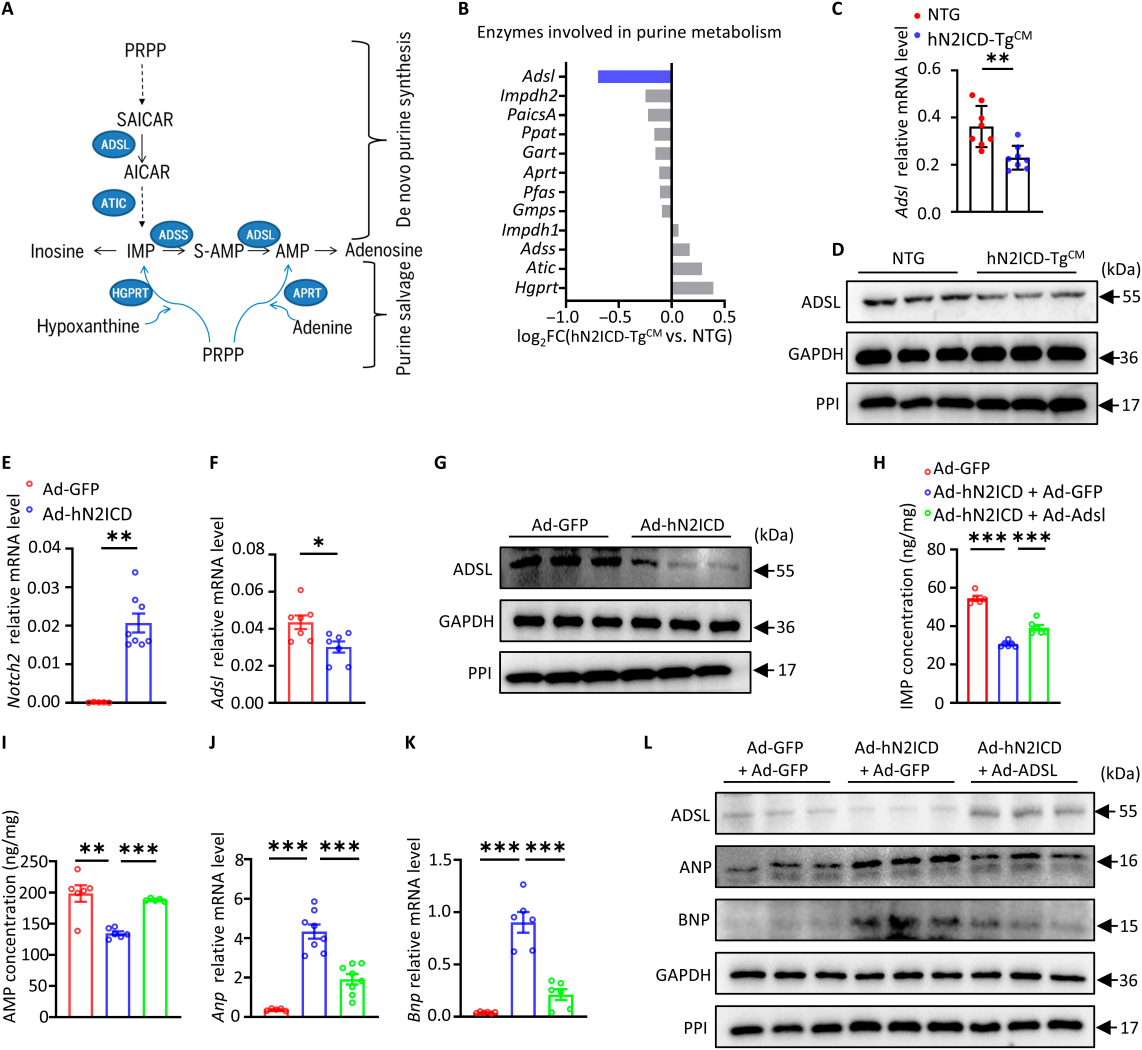
hN2ICD disrupts purine metabolism in cardiomyocytes by suppressing adenylosuccinate lyase (ADSL) expression. (A) Schematic overview of the major enzymes and metabolites involved in purine metabolism. (B) The enzymes involved in purine metabolism were identified by RNA sequencing (RNA-seq) in mouse heart tissue (hN2ICD-Tg^CM^ vs. NTG); ADSL is marked in blue. (C and D) Relative mRNA and protein levels of ADSL in mouse heart tissues. *n* = 6 to 8. (E and F) Relative mRNA levels of *hN2ICD* and *Adsl* expression in NRVMs with or without hN2ICD overexpression. *n* = 5 to 8. (G) Relative protein levels of ADSL in NRVMs with or without hN2ICD overexpression. (H and I) Intracellular IMP and AMP concentrations in hN2ICD-overexpressed NRVMs with or without ADSL re-expression. *n* = 5 and 6. (J and K) Relative mRNA levels of *Anp* and *Bnp* in hN2ICD-overexpressed NRVMs with or without ADSL re-expression. *n* = 5 to 8. (L) Western blot analysis of ADSL, ANP, and BNP proteins in hN2ICD-overexpressed NRVMs with or without ADSL re-expression. Data are shown as mean ± SEM. Statistical significance was evaluated with the Student *t* test (C and F), the Mann–Whitney *U* test (E), one-way ANOVA followed by Tukey’s multiple-comparisons test (H), and Brown–Forsythe and Welch one-way analysis (I, J, and K). SAICAR, succinyl aminoimidazole carboxamide riboside; AICAR, aminoimidazole carboxamide riboside; ADSS, adenylosuccinate synthetase; S-AMP, adenylosuccinate; HGPRT, hypoxanthine–guanine phosphoribosyltransferase; APRT, adenine phosphoribosyltransferase; ATIC, 5-aminoimidazole-4-carboxamide ribonucleotide formyltransferase/inosine monophosphate cyclohydrolase.

Purine metabolism includes the de novo purine biosynthetic and purine salvage pathway [21]. The de novo purine biosynthetic pathway consists of 10 highly conserved steps that transform phosphoribosyl pyrophosphate into IMP by 6 enzymes, which serves as precursors for the synthesis of AMP and guanosine 5′-monophosphate. Purine salvage can also generate IMP and guanosine 5′-monophosphate (Fig. 3A). Bulk RNA-sequencing analysis revealed that ADSL, which catalyzes the conversion of succinyl AICAR into AICAR along the de novo pathway and the conversion of adenylosuccinate (S-AMP) into AMP, was markedly decreased in hN2ICD-Tg^CM^ hearts compared to that in NTG hearts (Fig. 3B). Decreased ADSL expression in hN2ICD-Tg^CM^ hearts was further verified by real-time quantitative reverse transcription PCR and Western blot analyses (Fig. 3C and D). Consistently, hN2ICD overexpression suppressed ADSL expression in cultured NRVMs (Fig. 3E to G). More importantly, forced expression of ADSL in hN2ICD-overexpressed NRVMs restored intracellular IMP and AMP levels (Fig. 3H and I) and subsequently attenuated the hN2ICD-induced ANP and BNP expression (Fig. 3J to L). Thus, N2ICD drives cardiomyocyte hypertrophy by inhibiting ADSL-mediated biosynthesis of IMP and AMP.

### N2ICD represses ADSL transcription through HES1 in CMs

**Fig. 4.**
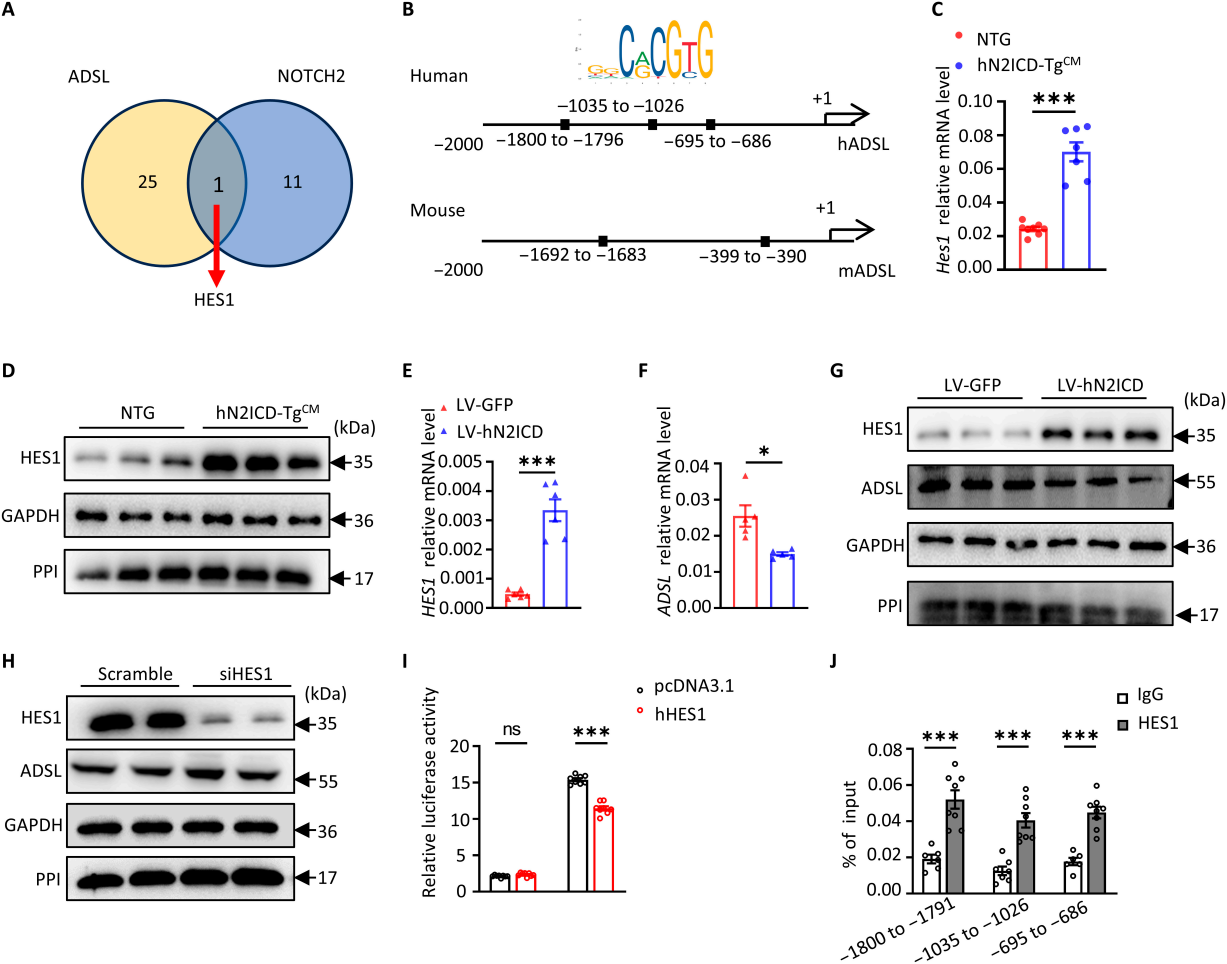
N2ICD inhibits ADSL transcription in cardiomyocytes through up-regulation of hairy and enhancer of split 1 (HES1). (A) Venn diagram showing the overlap of Notch target genes and transcription factors that regulate ADSL expression. (B) The binding sites of HES1 in the promoter region of human or mouse ADSL genes were predicted using JASPAR. (C) Relative mRNA levels of *Hes1* in the heart tissues of NTG and hN2ICD-Tg^CM^ mice. *n* = 7 and 8. (D) The protein levels of HES1 in heart tissues of NTG and hN2ICD-Tg^CM^ mice. (E and F) Relative mRNA levels of *HES1* and *ADSL* in AC16 cardiomyocytes with or without hN2ICD overexpression. *n* = 5 and 6. (G) Western blot analysis of HES1 and ADSL protein expression in AC16 cells with or without hN2ICD overexpression. (H) Western blot analysis of the protein expression of HES1 and ADSL in hN2ICD-overexpressed AC16 cells transfected with control or HES1 small interfering RNA (siRNA). (I) Luciferase reporter assay for the luciferase activity of the ADSL promoter in the presence of HES1 in HEK293T cells. *n* = 8. (J) Chromatin immunoprecipitation–quantitative polymerase chain reaction-quantitative polymerase chain reaction (ChIP–qPCR) analysis of HES1 enrichment at the human ADSL promoter in hN2ICD-overexpressed AC16 cells. *n* = 6 to 8. Statistical significance was evaluated using the Student *t* test for (I) and (J) and Welch’s *t* test for (C), (E), and (F). IgG, immunoglobulin G; hADSL, human adenylosuccinate lyase; mADSL, mouse adenylosuccinate lyase.

To investigate the mechanism underlying the reduced ADSL expression in N2ICD-overexpressed CMs, we predicted potential transcription factors (TFs) that bind to the promoter of ADSL using the online bioinformatics tool PROMO. By intersecting these TFs with the gene set of “Notch2 transcription” from the Reactome pathway database, we obtained one common TF: Hes1—a transcriptional repressor that belongs to the downstream target of Notch signaling (Fig. 4A). Motif analysis using JASPAR revealed multiple Hes1-binding sites in the Adsl promoter region of both humans and mice (Fig. 4B). Notably, the mRNA and protein levels of HES1 were significantly increased in hN2ICD-Tg^CM^ hearts (Fig. 4C and D), and overexpression of hN2ICD in AC16 cardiomyocytes markedly increased HES1 expression, subsequently leading to the down-regulation of ADSL expression (Fig. 4E to G). Moreover, ADSL expression increased after silencing HES1 in hN2ICD-overexpressed AC16 cells (Fig. 4H). Consistently, the dual-luciferase reporter gene assay and chromatin immunoprecipitation–quantitative PCR (ChIP–qPCR) showed that HES1 directly repressed *ADSL* transcription (Fig. 4I) and bound to the promoter region of *ADSL* (−1800 to −1791, −1035 to −1026, and −695 to −686) in AC16 cells (Fig. 4J).

### hN2ICD promotes CM hypertrophy through the AMPK/mTOR pathway

**Fig. 5.**
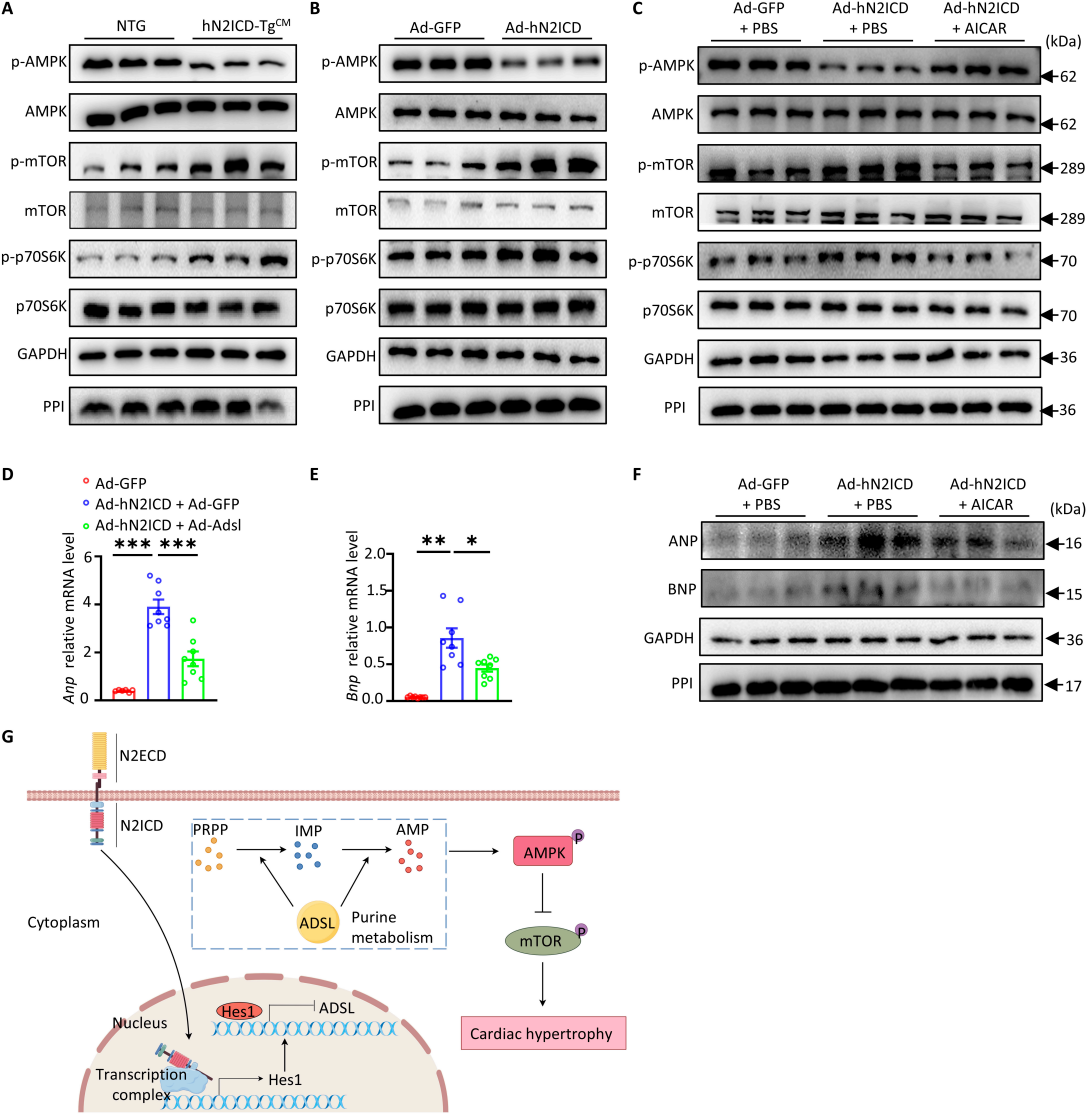
hN2ICD induces cardiomyocyte hypertrophy by relieving AMP-activated kinase (AMPK)-mediated inhibition of mammalian target of rapamycin (mTOR) signaling. (A and B) Western blot analysis of phosphorylated AMPK (p-AMPK), AMPK, phosphorylated mTOR (p-mTOR), mTOR, phosphorylated p70S6K (p-p70S6K), and p70S6K levels in heart tissues and NRVMs with or without hN2ICD overexpression. (C) Western blot analysis of p-AMPK, AMPK, p-mTOR, mTOR, p-p70S6K, and p70S6K levels in hN2ICD-overexpressed NRVMs with or without AICAR treatment. (D and E) Relative mRNA levels of *Anp* and *Bnp* in hN2ICD-overexpressed NRVMs with or without AICAR treatment. *n* = 6 to 8. (F) Western blot analysis of ANP and BNP protein levels in hN2ICD-overexpressed NRVMs with or without AICAR treatment. (G) Schematic illustration of Notch2/Hes1-derived cardiomyocyte hypertrophy through the ADSL/AMPK/mTOR pathway. Statistical significance was evaluated using Brown–Forsythe and Welch one-way analyses (D and E). N2ECD, Notch2 extracellular domain.

AMP is a highly conserved trigger of AMPK activation [22], and AMPK negatively regulates mTOR complex 1 activity, which is a key factor for cardiac hypertrophy [23]. Given the decreased AMP concentration in hN2ICD-Tg^CM^ hearts, we reasoned that low intracellular AMP levels may lead to compromised AMPK activity in these hearts. Indeed, AMPK activity was markedly reduced in hN2ICD-Tg^CM^ heart tissues, whereas the activities of mTOR and its downstream effector p70S6K were significantly enhanced (Fig. 5A). Moreover, in cultured hN2ICD-overexpressed NRVMs, robust mTOR/p70S6K activation and reduced AMPK activity were observed (Fig. 5B). Treatment with AICAR, an AMPK agonist, abrogated the enhanced mTOR activity by restoring AMPK activation in hN2ICD-overexpressed NRVMs (Fig. 5C) and subsequently attenuated the hN2ICD-driven increase in ANP and BNP expression (Fig. 5D to F). Thus, activation of Notch2 promoted CM hypertrophy through the HES1/ADSL/AMPK/mTOR signaling pathway (Fig. 5G).

### Administration of AICAR alleviates cardiac hypertrophy and dysfunction in hN2ICD-Tg^CM^ mice

**Fig. 6.**
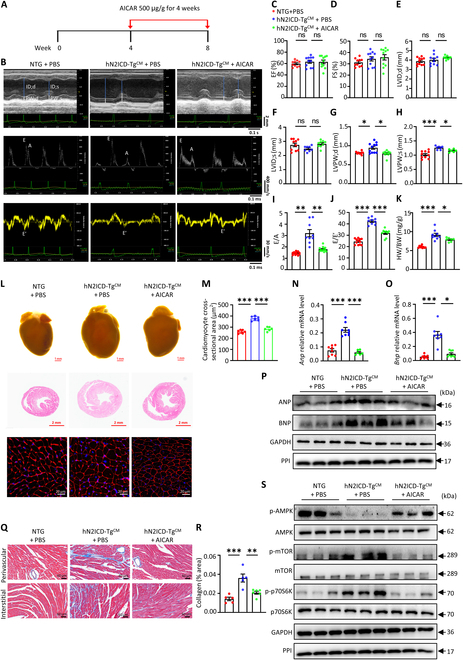
Aminoimidazole carboxamide riboside (AICAR) treatment improves the impaired cardiac function in hN2ICD-Tg^CM^ mice. (A) Schematic diagram of AICAR administration to hN2ICD-Tg^CM^ mice. (B) Representative echocardiographic tracings of hN2ICD-Tg^CM^ mice with or without AICAR treatment. (C) Percentage of LVEF. *n* = 10 and 11. (D) Percentage of LVFS. *n* = 10 and 11. (E and F) Quantitative analysis of LVID;d and LVID;s obtained from echocardiography. *n* = 8 to 11. (G and H) Quantitative analysis of LVPW;d and LVPW;s. *n* = 6 to 11. (I) Ratio between E (early filling) and A (atrial filling) waves. *n* = 9 and 13. (J) Ratio between mitral E and E′ wave (E/E′). *n* = 9 and 12. (K) HW/BW ratio of mice. *n* = 7 to 9. (L) Representative images of whole-mount hearts and H&E- and WGA-stained section of hearts from mice. Scale bars, 1 mm (general view), 2 mm (H&E), and 20 μm (WGA). *n* = 6 and 7 mice. (M) Quantitative analysis of WGA staining. *n* = 6 per group. (N and O) Relative mRNA levels of *Anp* and *Bnp* in mouse heart tissues. *n* = 6 to 8. (P) Western blot analysis of ANP and BNP protein levels in mouse heart tissues. (Q) Representative images of Masson’s trichrome staining of cardiac tissues. Scale bar, 60 μm. *n* = 5 and 6. (R) Quantification of collagen content in mouse heart tissues. (S) Western blot analysis of p-AMPK, AMPK, p-mTOR, mTOR, p-p70S6K, and p70S6K protein levels in mouse heart tissues. Statistical significance was evaluated using one-way ANOVA followed by Tukey’s multiple-comparisons test (C, D, F, J, M, N, and R) and Brown–Forsythe and Welch one-way analyses (E, G, H, I, and K) and Kruskal–Wallis test (O).

To investigate whether the restoration of AMPK activity could rescue cardiac dysfunction in hN2ICD-Tg^CM^ mice, we treated 4-week-old hN2ICD-Tg^CM^ mice with AICAR for 4 weeks (Fig. 6A). Strikingly, AICAR had no obvious effect on EF, FS (Fig. 6B to D), and ventricular systolic function (LVID;d and LVID;s) (Fig. 6E and F) but significantly decreased ventricular wall thickness (LVPW;d and LVPW;s) in hN2ICD-Tg^CM^ hearts (Fig. 6G and H) and improved left ventricular diastolic function as evidenced by reduced E/E′ and E/A ratios in AICAR-treated mice compared to those in phosphate-buffered saline (PBS)-treated NTG mice (Fig. 6I and J). In line with these results, AICAR markedly attenuated cardiac hypertrophy, as indicated by the reduced HW/BW ratio (Fig. 6K), the decreased heart size and CM surface area (Fig. 6L and M), and reduced BNP and ANP expression (Fig. 6N to P) in hN2ICD-Tg^CM^ mice. Moreover, the increased myocardial fibrosis in hN2ICD-Tg^CM^ mice was significantly reduced by AICAR administration (Fig. 6Q and R). In agreement with these observations, AICAR treatment increased AMPK activity and suppressed mTOR/p70S6K signaling in hN2ICD-Tg^CM^ heart tissues (Fig. 6S). Thus, targeting AMPK activity may have therapeutic potential for the treatment of Notch2 overactivation-induced cardiac dysfunction.

### Gain-of-function mutation of Notch2 leads to pathological hypertrophy in human cardiomyocytes

**Fig. 7.**
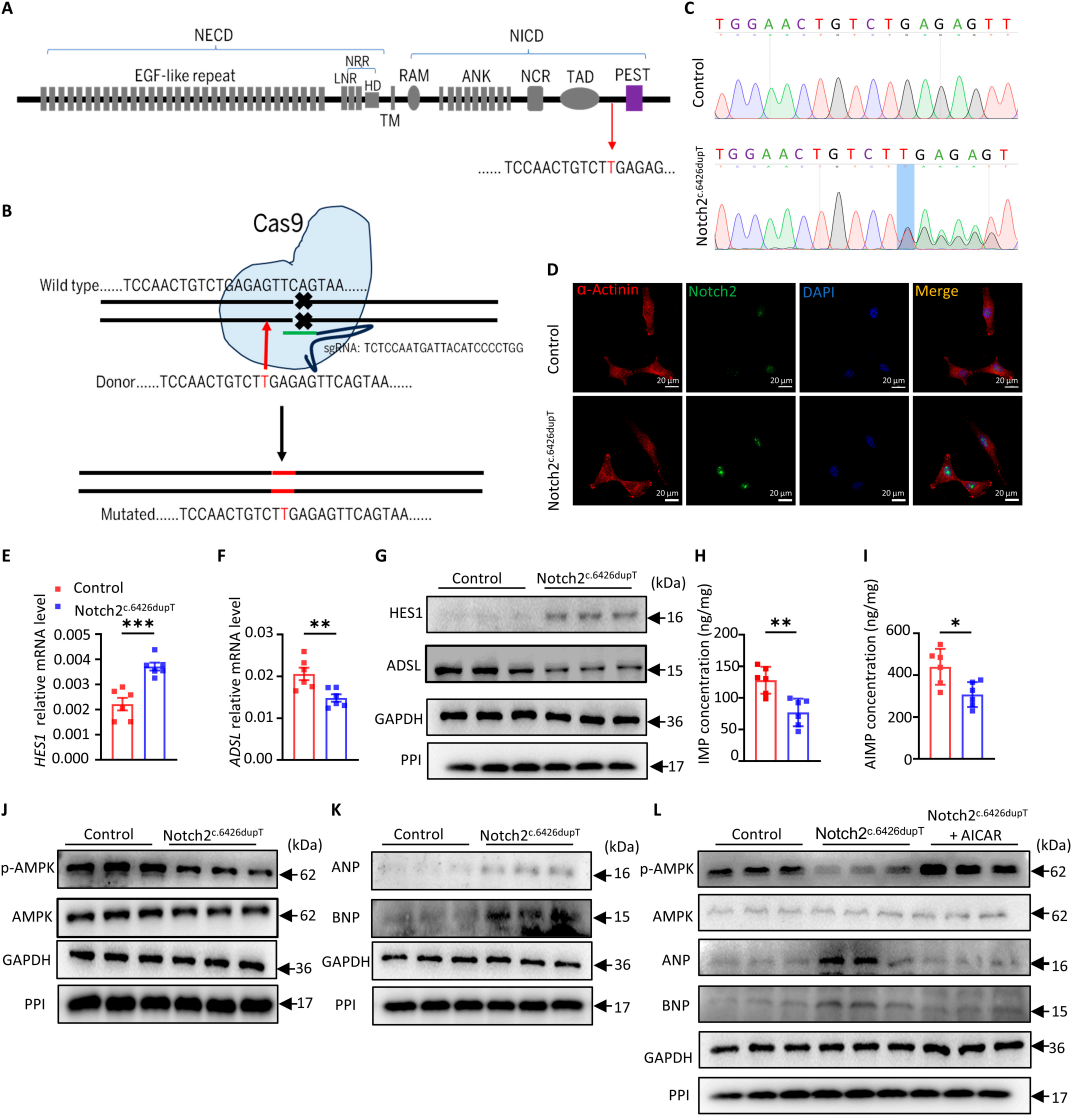
Gain-of-function of Notch2 promotes pathological hypertrophy in human cardiomyocytes. (A) Schematic diagram of a frameshift mutation of the Notch2 proline, glutamic acid, serine, and threonine (PEST) region (c.6426dupT) from Hajdu–Cheney syndrome (HCS) patients. Thymine (T) is inserted at position 6426 of the Notch2 gene. (B) Strategy diagram of the construction of AC16 cells harboring the c.6426dupT mutation by the CRISPR/CRISPR-associated protein 9 (CRISPR/Cas9) method. (C) Chromatograms of Notch2 mutations identified in AC16 cells by DNA sequencing. (D) Immunofluorescence showing the distribution and expression of Notch2 in AC16 cells. (E to G) Relative mRNA and protein levels of HES1 and ADSL in control and Notch2^c.6426dupT^ AC16 cells. *n* = 6. (H and I) Intracellular IMP and AMP concentrations in control and Notch2^c.6426dupT^ AC16 cells. *n* = 6. (J and K) Western blot analysis of p-AMPK, AMPK, ANP, and BNP protein levels in control and Notch2^c.6426dupT^ AC16 cells. (L) Western blot analysis of the protein levels of p-AMPK, AMPK, ANP, and BNP in Notch2^c.6426dupT^ AC16 cells with or without AICAR treatment. Statistical significance was evaluated using the Student *t* test (E, F, H, and I). EGF-like, epidermal growth factor-like; NRR, negative regulatory region; LNR, LIN12–Notch repeats; HD; heterodimerization domain; RAM, RBPjκ association module; TM, transmembrane domain; ANK, ankyrin repeats; NCR, Notch cytokine response region; TAD, transcriptional activation domain; sgRNA, single guide RNA; DAPI, 4′,6-diamidino-2-phenylindole.

A heterozygous frameshift mutation in exon 34 of Notch2 (c.6426dupT) has been found in patients with HCS, resulting in severe congenital heart disease in these patients [24]. This mutation disrupts the PEST domain and leads to sustained N2ICD activity by the blockage of its ubiquitin-mediated degradation [25]. To investigate the impact of this gain-of-function mutation on human cardiomyocytes, we generated a c.6426dupT mutation in Notch2 gene using the CRISPR/CRISPR-associated protein 9 (CRISPR/Cas9) system in AC16 human cardiomyocytes (Fig. 7A to C). Notably, the c.6426dupT mutation led to N2ICD accumulation in the nucleus of AC16 cells (Fig. 7D). Notch2^c.6426dupT^ cells essentially recapitulated the phenotype observed in hN2ICD-overexpressed CMs, displaying enhanced HES1 expression, reduced ADSL expression (Fig. 7E to G), lower intracellular IMP and AMP levels (Fig. 7H and I), reduced AMPK activity (Fig. 7J), and elevated ANP and BNP expression compared to control cells (Fig. 7K). Again, AICAR treatment effectively increased AMPK activity and suppressed ANP and BNP expression in Notch2^c.6426dupT^ cells (Fig. 7L).

## Discussion

In this study, we found that overexpression of hN2ICD in cardiomyocytes leads to left ventricular diastolic dysfunction with a preserved EF and cardiac hypertrophy in mice. hN2ICD overexpression induces cardiomyocyte hypertrophy by compromising purine nucleotide metabolism. We identified that ADSL, an enzyme for purine biosynthesis, is down-regulated in cardiac tissues in hN2ICD-Tg^CM^ mice, accompanied with suppressed mTOR activity and increased AMPK activity. Administration of the AMPK agonist AICAR ameliorates cardiac dysfunction in hN2ICD-Tg^CM^ mice. These results lend credence to the hypothesis that purine metabolism is essential for Notch2-derived cardiac hypertrophy and dysfunction.

HCS is a rare genetic disorder with characteristic osteolysis; cardiac defects are also known associated abnormalities [2]. It is caused by gain-of-function mutation of Notch2 gene. Forced expression of the mouse N2ICD fragment, specifically in the myocardium, leads to hypertrabeculation and increased heart mass during cardiac development in mice, resulting in embryonic lethality [10]. In culture, we observed that gain-of-function mutation of Notch2 gene (c.6426dupT) leads to enlargement of human cardiomyocytes. Using a humanized mouse expressing the human N2ICD fragment in cardiomyocytes, we unveiled Notch2 overactivation results in cardiac hypertrophy, left ventricular diastolic dysfunction in mice with preserved EF. Thus, we speculate that cardiac hypertrophy may play a role in the development of congestive heart failure in patients with HCS, along with cardiac developmental defects. In addition, Notch activation extends the duration of the action potential duration of adult myocytes, which may lead to bradycardia and increase patient mortality [26–28]. Moreover, inhibition of 4 different Notch receptors through the overexpression of the dominant-negative truncated form of mastermind-like protein in cardiomyocytes shortened the PR interval and increased the heart rates in 5-month-old mice [29]. Likewise, reduced heart rates were also observed in hN2ICD-Tg^CM^ mice. Thus, bradycardia may be also involved in impaired heart function in hN2ICD-Tg^CM^ mice. Overexpression of mouse N2ICD in cardiomyocytes causes embryonic lethality (E12.5 to E15.5) in mice [9]; however, almost 60% hN2ICD-Tg^CM^ mice survive after weaning. Protein alignment analysis reveals a total of 32-amino-acid difference in the transcriptional activation domain and PEST domain between mouse and human N2ICD fragments, indicating that human Notch2 may have a different transcriptional activity from mouse Notch2 [30,31].

Patients with hypertrophic cardiomyopathy (HCM) show higher expression of Notch2 and its target gene Hes1 in the left ventricle than patients with dilated cardiomyopathy [32]. Hes1 knockdown induces a dramatic reduction of hypertrophy in isoproterenol- or phenylephrine-treated NRVMs [33]. Overexpression of N2ICD in embryonic-stem-cell-derived and early neonatal cardiomyocytes triggers the hyperproliferation of working myocytes [34], supporting the crucial role of Notch2 in the proliferative expansion of the ventricular wall. In contrast, myocardial hypoplasia and reduced myocardial trabeculation have been observed in hypomorphic Notch2-mutant murine embryos [9]. Notably, the pro-ventricular hypertrophy effect of Notch2 is quite different from the functions of Notch1 and Notch3 in cardiac development, since mice lacking each of them develop HCM [35,36]. In addition, it appears that Notch signaling in cardiomyocytes is not necessary for the later phases of cardiac development [6,37]; activation of Notch in damaged heart improves cardiac functional performance and confers cardioprotection against cardiac injury [37,38]. High glucose levels induce Notch2 expression in endothelial cells and trigger cardiac fibrosis through endothelial–mesenchymal transition [39]. However, forced N2ICD overexpression in endothelial cells promotes apoptosis by decreasing survivin expression [40]. Interestingly, overexpression of the Notch ligand Jagged1 in cardiomyocytes suppressed myofibroblast proliferation in stressed adult hearts by cell communication [41].

The link between maladaptive left ventricular hypertrophy and diastolic dysfunction has long been established [42]. Hypertrophic cardiomyocytes undergo metabolic reprogramming in response to sustained stress. Diastolic dysfunction in hypertrophied cardiomyocytes is typically associated with reduced energy reserves [43–45]. AMPK serves as an energy sensor and has been shown to inhibit cardiac hypertrophy by negatively regulating mTOR-signaling-mediated protein synthesis [46]. AMPK activity had a significant decrease in the cardiac tissue of HFpEF mice compared with that in NTG mice [47]. Treatment with metformin, a potent AMPK activator, it markedly attenuated atrial fibrillation preponderance in HFpEF mice [47]. In line with these results, we noticed that AMPK activation significantly decreased along with enhanced mTOR signaling in N2ICD-overexpressed cardiomyocytes, and administration of the AMP mimetic AICAR confers cardioprotection against N2ICD-induced cardiac dysfunction. Suppression of AMPK activity by Notch2 signaling is also observed in retard osteoblast differentiation in HCS patients [48]. Cardiac adenine nucleotide depletion appears in both acute myocardial ischemia and chronic heart failure, which leads to reduced cardiac adenosine triphosphate (ATP) and induction of inorganic phosphate, thereby influencing cardiac mechanical work [49]. Our stable isotope tracer experiment demonstrated that N2ICD overexpression significantly reduced de novo IMP, AMP, adenosine diphosphate (ADP), and ATP production in ^15^N_2_-glutamine-incubated AC16 cardiomyocytes. Adenylate kinase gene expression was not notably altered in hN2ICD-Tg^CM^ heart tissues. Thus, human Notch2 intracellular domain overactivation depressed adenine nucleotide production may influence the cellular ATP:ADP ratio—a control parameter of energy metabolism [50], which, in turn, also contributes to the progression of impaired myocardial mechanical function and HCM [51]. Previous studies showed that ADSL modulates the AMP/ATP ratio and impacts AMPK activity, which has an impact on several important cellular processes [52–54]. Moreover, Notch signaling has also been linked to purine nucleotide biosynthesis [15]. A recent report demonstrated that adenylosuccinate synthase (Adssl1), which catalyzes the last step of the de novo purine synthesis pathway, promotes cardiomyocyte proliferation and heart regeneration after myocardial infarction [55], indicating that purine nucleotide metabolism may be targeted therapeutically to alleviate heart conditions. Further studies are warranted to confirm these findings in other preclinical cardiac dysfunction models and well-designed clinical studies.

In summary, gain-of-function mutations in Notch2 gene induce cardiac hypertrophy and diastolic dysfunction by disrupting ADSL-mediated AMP biosynthesis, and targeting purine nucleotide metabolism may represent an attractive therapeutic strategy for the treatment of cardiac diseases.

## Materials and Methods

### Reagents

AMP (No. 01930) and IMP (No. I4625) were purchased from Sigma-Aldrich. SimpleChIP Plus Enzymatic Chromatin IP Kit (Magnetic Beads) (No. 9005) was purchased from Cell Signaling Technology (CST). Luciferase Assay System (No. E1500) was purchased from Promega. Puromycin (No. HY-B1743A) was obtained from MedChemExpress. Dulbecco’s modified Eagle medium (DMEM) basic buffer (No. C11995500BT), fetal bovine serum (FBS; No. 16000-044), and penicillin/streptomycin dual antibiotics (No. 15140122) were purchased from Gibco, USA. Type II collagenase (No. LS004177) was purchased from Worthington.

### Animals

hN2ICD transgenic mice (hN2ICD-Tg^stop^) were generated using targeted conditional knock-in human Notch2 coding DNA sequence (amino acids 1699 to 2471) to mouse ROSA26 locus, containing the “CAG-loxP-Stop-loxP-polyA” cassette by Cyagen Biosciences (Suzhou, China). hN2ICD-Tg^CM^ mice were created by crossing hN2ICD-Tg^stop^ with αMHC^Cre^ mice [56]. hN2ICD-Tg^stop^ mice were utilized as littermate controls. The experiments were conducted using only male mice. Every mouse was of C57BL/6 genetic background and housed in specific-pathogen-free laboratory conditions. For AICAR treatment, 4-week-old hN2ICD-Tg^CM^ mice were randomly assigned to control and AICAR groups. AICAR mice were intraperitoneally injected with 0.5 mg/kg AICAR daily for 28 d [57]. Control mice were administered an equal volume of PBS. All animal experiments were performed in accordance with the approval of the Institutional Animal Care and Use Committee of Tianjin Medical University.

### Echocardiography and tissue Doppler imaging

A Vevo 2100 (Visual Sonics Inc., Toronto, Canada) fitted with an MS400 transducer was used to perform transthoracic echocardiography. Mice were anesthetized by inhalation of isoflurane (1% to 1.5%) throughout the procedure. Left ventricular systolic function was measured using parasternal long-axis M-mode scans. Left ventricular EF, left ventricular FS, LVID;d, LVID;s, LVPW;d, and LVPW;s were measured. Pulsed-wave and tissue Doppler imaging at the mitral valve level in the apical 4-chamber view were used to evaluate left ventricular diastolic function. The peak Doppler blood inflow velocity across the mitral valve during early diastole (E), peak Doppler blood inflow velocity across the mitral valve during late diastole (A), and peak tissue Doppler myocardial relaxation velocity at the mitral valve annulus during early diastole (E′) were measured, and E/A and E/E′ were calculated.

### Histological analysis

The hearts of mice were removed and fixed for 48 h in 4% paraformaldehyde. As previously mentioned, heart tissues were embedded, sectioned, and stained using hematoxylin and eosin, wheat germ agglutinin, and Masson’s trichrome staining [56]. Wheat germ agglutinin images were captured with a confocal laser scanning microscope (Carl Zeiss, Oberkochen, Germany), the cross-sectional size of cardiomyocytes was measured using the ImageJ software (National Institutes of Health, USA), and at least 100 cells per sample were measured [58].

### Immunofluorescence staining

Cell-climbing slices or frozen sections containing cardiac tissue were fixed in cold acetone and blocked with 3% bovine serum albumin for 30 min to prevent nonspecific binding. Slices were then incubated with primary antibodies overnight, as follows: anti-α-actinin (1:200, Catalog [Cat.] No. A7811, Sigma-Aldrich) and anti-Notch2 (1:2,000, Cat. No. 4530, CST). Then, they were incubated with secondary antibodies (Alexa Fluor 488–anti-rabbit and Alexa Fluor 594–anti-mouse, 1:1,000, Invitrogen) for 2 h. 4′,6-Diamidino-2-phenylindole (Invitrogen) was used to counterstain the slides, the cover glass was mounted, and the tissue was visualized.

### Lentivirus construction

The complementary DNA (cDNA) of hN2ICD (amino acids 1699 to 2471) was subcloned into the lentiviral vector PCDH-PURO (Hanheng Biotechnology). hN2ICD, psPAX2, and pMD2G vectors were mixed at a ratio of 4:3:1 and co-transfected into 293T cells using EL Transfection Reagent (TransGen). Viral supernatants were collected and then filtered. The samples were then centrifuged with an ultracentrifuge at 28,000 g for 2 h at 4 °C; after centrifugation, the supernatant could be discarded and the virus was resuspended in PBS. The viruses were stored at −80 °C.

### Adenovirus construction

Relative adenoviruses were constructed by Hanheng Biotechnology. Briefly, cDNAs of ADSL and hN2ICD (amino acids 1699 to 2471) were subcloned into the Ad5 adenoviral shuttle vector, respectively. Adenoviruses (AdMax Adenoviral Expression System) were propagated in HEK293A cells. The titer of stocks used for these studies measured by plaque assays was 2.5 × 10^10^ plaque-forming units (pfu)/ml. All viruses were stored at −80 °C.

### Isolation and culture of primary NRVMs

NRVMs were isolated from the ventricles of 1- to 3-d-old Sprague–Dawley rat pups and cultured as previously described [59]. In brief, the hearts were obtained from neonatal rats, the atria were removed, the ventricles were cut up and digested several times at 37 °C with the digestive solution (0.5 mg/ml type II collagenase in Hank’s balanced salt solution), cells were filtered with a 100-μm cell filter, and the cells were eventually collected by centrifugation. Since fibroblasts adhere to the dish faster than cardiomyocytes, fibroblasts were therefore excluded by differential adherent separation, and ventricular myocytes were obtained and cultured in complete medium (10% FBS and 1% penicillin/streptomycin in DMEM basic medium). Serum-free medium was used 24 h later. After overnight serum starvation, NRVMs were infected with hN2ICD adenovirus (2.5 × 10^7^ pfu/ml) for 48 h. For rescue experiments, NRVMs overexpressing hN2ICD were treated with AMP (40 μM) and AICAR (200 μM) for 24 h or infected with ADSL adenovirus for 36 h, respectively.

### Generation of a stable cell line with the Notch2 point mutant

To create a stable cell line with the Notch2 point mutant, guide RNA (gRNA) (TCTCCAATGATTACATCCCCTGG) was cloned into the vector (pHBLV-CAS9-PURO), and the donor (single-nucleotide polymorphism sequence containing an insertional mutation at c.6426dupT) [24] was cloned into the vector (PCDH-neo) by Hanheng Biotechnology. The gRNA or donor was mixed with psPAX2 and pMD2G at a ratio of 4:3:1 and co-transferred into 293T cells using EL Transfection Reagent (TransGen). The viral supernatant was collected, filtered, centrifuged, and resuspended, as described above, for lentivirus construction. AC16 cells were infected with gRNA and the donor virus for 6 h and cultured, and then the cells were screened by using puromycin (2 μg/ml) and G418 (4 μg/ml) for 72 h. The surviving cells were identified as Notch2 point mutant cells (Notch2^c.6426dupT^). To verify whether the point mutant cells were successfully constructed, DNA was extracted from the mutant cells and subjected to Sanger sequencing. The sequencing primers were as follows: F—CTCTCTCACCTGTCATCT; R—GCATAACTGTGCTGTGAA.

### Western blotting

Tissues and cells were lysed with lysates (containing cocktail), protein concentrations were determined, and the proteins were denatured, separated, and transferred onto polyvinylidene fluoride membranes (Millipore). Membranes were incubated sequentially with 5% skim milk for 2 h, primary antibody at 4 °C overnight, and secondary antibody for 2 h. The primary antibodies used were as follows: anti-Notch2 (1:1,000, Cat. No. 4530, CST), anti-Hes1 (1:1,000, Cat. No. 11988, CST), anti-P-AMPK (1:1,000, Cat. No. 2535, CST), anti-AMPK (1:1,000, Cat. No. 2532, CST), mTOR (1:1,000, Cat. No. 2983, CST), anti-phospho-mTOR (Ser2448) (1:1,000, Cat. No. 5536T, CST), anti-p70 S6 kinase (1:1,000, Cat. No. 2708T, CST), anti-phospho-p70 S6 kinase (Thr421/Ser424) (1:1,000, Cat. No. 9204, CST), anti-ANP (1:1,000, Cat. No. A22075, ABclonal), anti-BNP (1:1,000, Cat. No. A2179, ABclonal), and anti-ADSL (1:500, Cat. No. 15264-1-AP, Proteintech). Blots were scanned using a Tanon Imaging System (Tanon-5200Multi; Shanghai, China).

### Real-time quantitative reverse transcription PCR

Total RNA was isolated. Next, cDNA was synthesized using cDNA Synthesis Kit (Cat. No. 11141ES60, Yeasen), and cDNA was amplified. The relative expression of the target gene was displayed after being normalized to that of the internal control. The primers used for real-time quantitative reverse transcription PCR are listed in Table S1.

### Luciferase assay

The human ADSL promoter sequence (−2000 to +1) was cloned into the PGL3-Basic vector to generate PGL3-ADSL-promoter (Hanheng Biotechnology). The coding DNA sequence of human HES1 was cloned into the pcDNA3.1 vector (Hanheng Biotechnology). The HEK293T cells were transfected as indicated with the PGL3-ADSL-promoter, pcDNA3.1-HES1, pcDNA3.1, and *Renilla* luciferase reporter genes (Promega). After 36 h, the cells were collected, and a luciferase assay system (Promega) was used to measure the firefly and *Renilla* luciferase activities in the cell lysates. Firefly luciferase activity was normalized to *Renilla* luciferase activity.

### ChIP–qPCR

A ChIP–qPCR assay was performed following the manufacturer’s protocol (SimpleChIP Plus Enzymatic Chromatin IP Kit) to detect endogenous binding between HES1 and ADSL promoter regions. AC16 cells overexpressing N2ICD were gathered and subjected to 1% formaldehyde, followed by the addition of 2.5 M glycine to cross-link chromatin. Nuclear pellets were sonicated after being resuspended in ChIP buffer. The nuclear pellet suspension was incubated with anti-HES1 antibody (1:50, Cat. No. 11988, CST) for an entire night at 4 °C. DNA purification beads were used to purify the DNA–protein complexes, and elution buffer was used to elute them. The purified immunoprecipitated DNAs were subjected to determination of ADSL gene expression by qPCR, and relative occupancies were normalized to the input DNA (sequences are shown in Table S2).

### Cell culture

Human AC16 cardiomyocytes and HEK293T cells were cultured in DMEM (Cat. No. C11995500BT; Gibco) complete medium containing 10% FBS (Cat. No. 1600044; Gibco) and 1% penicillin/streptomycin (Cat. No. 15240062; Gibco). AC16 cells were infected with thegreen fluorescent protein (GFP) control or the hN2ICD lentivirus for 24 h. Stably infected AC16 cells were selected by incubation with puromycin (2 μg/ml) for 7 d, while cells that did not express hN2ICD were excluded under puromycin stress. To knock down HES1, hN2ICD-overexpressed AC16 cells were transfected with HES1 small interfering RNA (Table S3).

### RNA sequencing and analysis

RNA sequencing and data analyses were performed by Novogene. Total RNA was extracted from mouse heart tissue. Library preparations were sequenced and analyzed as described previously [60,61]. A corrected *P* value of 0.05 and an absolute fold change of 2 were set as the thresholds for significantly differential expression. All genes related to purine metabolism were identified. The raw RNA sequencing data in this paper were deposited in the National Center for Biotechnology Information (NCBI), under this accession number: PRJNA1147122.

### Untargeted metabolomics

Fresh hearts (6 samples per group) from NTG and hN2ICD-Tg^CM^ mice were rinsed with PBS. Metabolites were extracted and detected as described in the literature [62]. After normalization to the total peak intensity, the processed data were subjected to multivariate data analysis, including Pareto principal component analysis and orthogonal partial least squares DA. Each variable’s contribution to the classification was ascertained by calculating its variable importance in projection value in the orthogonal partial least squares DA model. An enrichment analysis was carried out to investigate the effects of metabolites that were expressed differently. KEGG pathway enrichment analyses were conducted using Fisher’s exact test. Pathways were deemed significantly altered only with their *P* values under a threshold of 0.05. Raw data are available in MetaboLights at https://www.ebi.ac.uk/metabolights/, reference no. MTBLS10874.

### Analysis of purine metabolites

AMP and IMP production in cardiac tissues or cultured cells was quantitated by LC–MS/MS. The samples were separated on an ACQUITY UPLC@HSS T3 column (1.8 μm, 2.1 ∗ 100 mm, Waters, USA). The standards of AMP and IMP were dissolved and diluted in gradients in triple-distilled water, and standard curves were drawn. Each experimental group contained 6 samples (50 mg of mice heart, 5 × 10^6^ rat cardiomyocytes, or 10^7^ human cardiomyocytes). Each sample was mixed with 401 μl of pre-cooled extraction reagents (400 μl of 80% methanol + 1 μl of ATP isotope internal reference for each sample) as described previously [63]. Then, each sample was lysed 3 times on ice for 3 s each time with an ultrasonic crusher (hearts were ground with a tissue grinder). Next, 1 ml of methyl *tert*-butyl ether was added and vortexed for 10 min; 250 μl of triple-distilled water was added to the samples, which were then placed for 5 min at room temperature. Next, the mixtures were centrifuged at 12,000 rpm for 10 min at 4 °C. The lower aqueous phase was discarded, the residue was filtered with filter column, and the metabolite content was obtained after processing. The cell sediment was removed, and the protein content was measured. All samples were analyzed using a mass spectrometer. MS/MS was performed with the following transitions: *m*/*z* 348.1 (AMP) → *m*/*z* 136.1, *m*/*z* 347 (IMP) → *m*/*z* 79. The normalized peak areas of AMP and IMP were quantified with MultiQuant 3.0 (AB Sciex) according to the standard curve of the corresponding standard samples. The AMP and IMP contents were normalized to the protein content of the corresponding samples.

### Stable isotope labeling and metabolite profiling for targeted flux analyses

AC16 cells were spread in a 10-cm dish and treated for 15 h in the absence of serum. For ^5^N_2_-glutamine (amide-labeled) flux studies, AC16 cells were covered with glutamine-free DMEM for 2 h and then incubated medium containing 4 mM ^15^N_2_-glutamine (Cat. No. HY-N0390S8, MedChemExpress) for another 4 h. Metabolites were extracted as described in the protocol by Ali et al. [19]. Cell metabolites’ extraction, separation, and identification were performed by Shanghai ProfLeader Biotech Co., Ltd.

### Statistical analysis

All data are shown as mean ± standard error of the mean (SEM). Data were analyzed using GraphPad Prism 8 (GraphPad Prism Software Inc., San Diego, CA, USA). Data distribution was evaluated using the Shapiro–Wilk normality test; *P* > 0.05 indicates that variables followed a normal distribution in the population. Normally distributed data with equal variance were analyzed using the Student *t* test (2 groups) and one-way analysis of variance (ANOVA) with Tukey’s multiple-comparisons test (3 groups). Welch’s *t* test (2 groups) and Brown–Forsythe and Welch one-way analyses (3 groups) were used for data with unequal variance. The Mann–Whitney *U* test (2 groups) and Kruskal–Wallis test (3 groups) were used to compare the differences between samples when the sample distributions were not normally distributed. A value of *P* < 0.05 was considered a statistically significant difference. *P < 0.05; **P < 0.01; ***P < 0.001.

## Data Availability

All data are available from the corresponding authors upon request.
