# Supplementary material for: Notch2 Signaling Drives Cardiac Hypertrophy by Suppressing Purine Nucleotide Metabolism
Source: Research (Wash D C). 2025 Mar 18;8:0635. doi: 10.34133/research.0635 (PMC11913782; doi:10.34133/research.0635)
Supplement: Supplementary 1 — Fig. S1 Tables S1 to S3 [file research.0635.f1.docx]

Supplementary Materials for

Notch2 Signaling Drives Cardiac Hypertrophy by Suppressing Purine Nucleotide Metabolism

Yuhong Wang^1†^, Yizhe Li^1†^, Shihong Chen^1^, Tingting Yu^1^, Weiyan Sun^1^, Jiao Liu^1^, Huiwen Ren^1^, Yao Zhou^1^, Lu Wang^1^, Xixi Tao^2^, Ronglu Du^1^, Wenlong Shang^1^, Yinxiu Li^1^, Danyang Tian^1^, Bei Wang^1^, Yujun Shen^1^, Qian Liu^1^*, Ying Yu^1^*.

*Corresponding author. E-mail: [yuying@tmu.edu.cn](mailto:yuying@tmu.edu.cn) or bioliuqian@tmu.edu.cn.

**This file includes:**

Supplementary Figures S1

Supplementary Tables S1-3

**
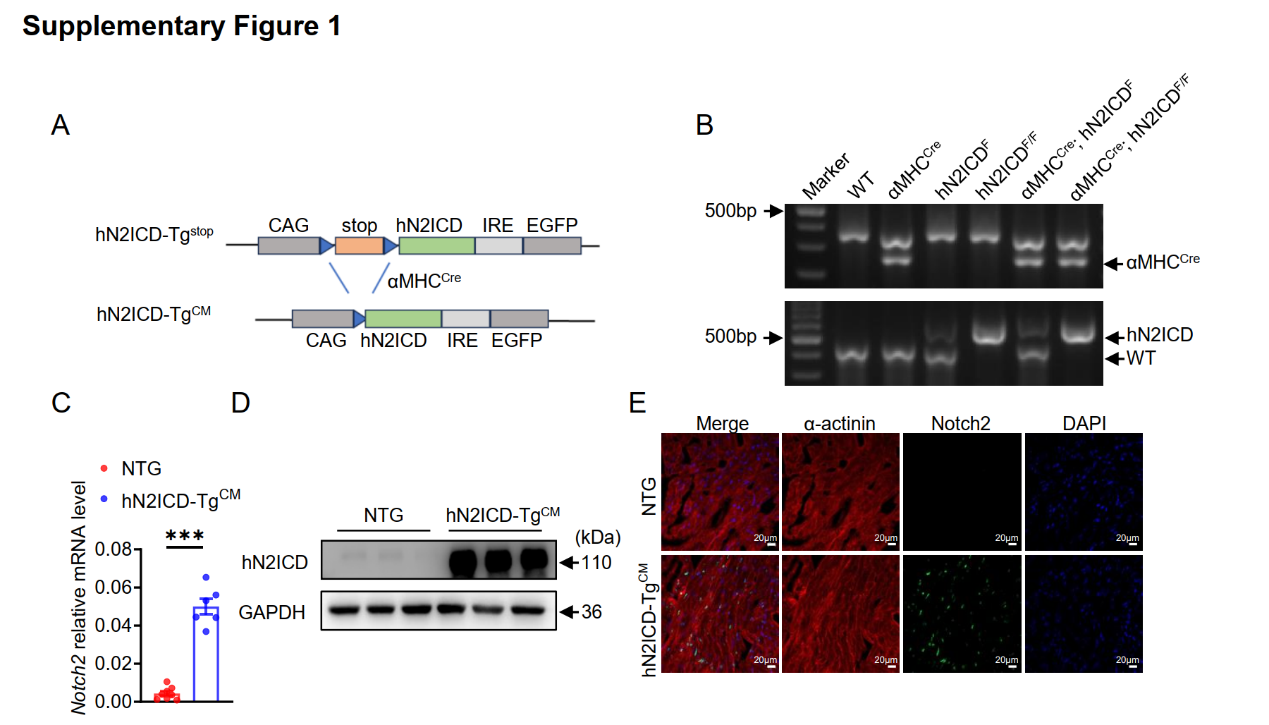
**

**Figure S1. Generation of hN2ICD-Tg^CM^ mice.** A. Schematic representation of the targeting strategy for generating cardiac hN2ICD transgenic mice. B. Genotyping of hN2ICD-Tg^CM^ and their littermate control mice. C-D. Relative N2ICD mRNA and protein levels in heart tissues from NTG and hN2ICD-Tg^CM^ mice. n=6-8. E. Immunofluorescence showing the distribution and expression of Notch2 in cardiomyocytes of mice. n=4-5. Data are shown as mean ± SEM. Statistical significance was evaluated using Student’s t-test (C). ***, P < 0.001.

**Table S1. Human, mouse, and rat oligonucleotide primers for RT-qPCR.**

| Species | Gene | Forward primer | Reverse primer |
| --- | --- | --- | --- |
| Human | ADSL | GCTGGAGGCGATCATGGTTC | TGATAGGCAAACCCAATGTCTG |
| Human | NOTCH2 | ACACGACACCGGATAAACCA | ATGCCGCGAGCTATCTTTCT |
| Human | HES1 | ACACGACACCGGATAAACCA | ATGCCGCGAGCTATCTTTCT |
| Human | GAPDH | GGAGCGAGATCCCTCCAAAAT | GGCTGTTGTCATACTTCTCATGG |
| Mouse | Anp | CGGAGCCTACGAAGATCCAG | AAGCTGTTGCAGCCTAGTCC |
| Mouse | Bnp | GAGGTCACTCCTATCCTCTGG | GCCATTTCCTCCGACTTTTCTC |
| Mouse | Hes1 | CCAGCCAGTGTCAACACGA | AATGCCGGGAGCTATCTTTCT |
| Mouse | Adsl | AGCCGCGAGATGTGTTTCTT | TCAATGTTGTTCAGGTTCGACTT |
| Mouse | Gapdh | AGGTCGGTGTGAACGGATTTG | TGTAGACCATGTAGTTGAGGTCA |
| Rat | Anp | GAAGATGCCGGTAGAAGATGAG | AGAGCCCTCAGTTTGCTTTTC |
| Rat | Bnp | GGTGCTGCCCCAGATGAT | CTGGAGACTGGCTAGGACTTC |
| Rat | Adsl | CTTGAAGCGTGTCCGAGATGA | ACTGGAACCAATCTGCTGCTTC |
| Rat | Gapdh | GACATGCCGCCTGGAGAAAC | AGCCCAGGATGCCCTTTAGT |

**Table S2. Oligonucleotide primers used for ChIP-qPCR**

| Species | Transcription factor binding site | Forward primer | Reverse primer |
| --- | --- | --- | --- |
| Human | -695~-686 | ACAGGTACAGGCCG | GGAGGTCGAGGCAGG |
| Human | -1035~-1026 | CTCAGGCTGGTCTCCAA | GACAGGGTCTTGCTA |
| Human | -1800~-1791 | CTGGAGTGCAGTGGCA | GGTGGATCACCTGAG |

**Table S3. siHES1**

| siRNA | Forward primer | Reverse primer |
| --- | --- | --- |
| siHES1-1 | CAACACGACACCGGAUAAATT | UUUAUCCGGUGUCGUGUUGTT |
| siHES1-2 | GAGCACAGAAAGUCAUCAATT | UUGAUGACUUUCUGUGCUCTT |
| siHES1-3 | GAUGCUCUGAAGAAAGAUATT | UAUCUUUCUUCAGAGCAUCTT |
